# A Comparison of Two Multi-Tasking Approaches to Cognitive Training in Cardiac Surgery Patients

**DOI:** 10.3390/biomedicines11102823

**Published:** 2023-10-18

**Authors:** Irina Tarasova, Olga Trubnikova, Irina Kukhareva, Irina Syrova, Anastasia Sosnina, Darya Kupriyanova, Olga Barbarash

**Affiliations:** Department of Clinical Cardiology, Research Institute for Complex Issues of Cardiovascular Diseases, Sosnovy Blvd., 6, 650002 Kemerovo, Russia; truboa@kemcardio.ru (O.T.); syrova@kemcardio.ru (I.S.);

**Keywords:** cognitive training, multi-tasking, postoperative cognitive dysfunction, cardiac surgery

## Abstract

Background: The multi-tasking approach may be promising for cognitive rehabilitation in cardiac surgery patients due to a significant effect on attentional and executive functions. This study aimed to compare the neuropsychological changes in patients who have undergone two variants of multi-tasking training and a control group in the early postoperative period of coronary artery bypass grafting (CABG). Methods: One hundred and ten CABG patients were divided into three groups: cognitive training (CT) I (a postural balance task with mental arithmetic, verbal fluency, and divergent tasks) (*n* = 30), CT II (a simple visual–motor reaction with mental arithmetic, verbal fluency, and divergent tasks) (*n* = 40), and control (*n* = 40). Results: Two or more cognitive indicators improved in 93.3% of CT I patients, in 72.5% of CT II patients, and in 62.5% of control patients; CT I patients differed from CT II and control (*p* = 0.04 and *p* = 0.008, respectively). The improving short-term memory and attention was found more frequently in the CT I group as compared to control (56.7% vs. 15%; *p* = 0.0005). The cognitive improvement of all domains (psychomotor and executive functions, attention, and short-term memory) was also revealed in CT I patients more frequently than CT II (46.7% vs. 20%; *p* = 0.02) and control (46.7% vs. 5%; *p* = 0.0005). Conclusions: The CT I multi-tasking training was more effective at improving the cognitive performance in cardiac surgery patients as compared to CT II training and standard post-surgery management. The findings of this study will be helpful for future studies involving multi-tasking training.

## 1. Introduction

It is known that cardiovascular disease is a significant pathophysiological background for the development of ischemic brain damage [1,2,3]. Vascular changes have been shown to cause brain tissue pathology well in advance of clinical manifestations of neurological or cognitive deficits [4,5,6]. Currently, cognitive deficit is considered as a marker not only of low quality of life in patients, but also of a decrease in life expectancy. The deterioration of cognitive health contributes to a decrease in the patient’s adherence to treatment, which, in turn, can lead to the progression and worsening of the prognosis of cardiovascular disease [7]. Patients with cardiovascular disease and cognitive deficit who undergo cardiac surgery represent a distinct cohort that is difficult to manage [8,9]. Cardiopulmonary bypass surgery also contributes to an acute ischemic brain injury [10,11]. The most significant complications of cardiac surgery are stroke and postoperative cognitive deficit or dysfunction [POCD]. The prevalence of POCD is high and can reach 70% [12,13]. POCD development in cardiac surgery patients is associated with prolonged intensive care and hospital stay, as well as deterioration of rehabilitation procedures, ultimately reducing the effectiveness of surgery.

Currently, there are no clear approaches to POCD prevention and cognitive rehabilitation of cardiac surgery patients. High medical and social significance of POCD determines the need to develop new strategies for cognitive rehabilitation in cardiovascular disease patients that may preserve the quality of life and social status. Among nonpharmacological treatments for cognitive deficits, combined programs of physical and cognitive training are now becoming more widespread [14,15,16]. Recent studies show that physical and cognitive development are interdependent and closely related [16,17]. It has been established that neurogenesis continues even in adulthood [18], and physical activity is a key factor in neurogenesis, depending on the intensity and systemic effect [19]. At the same time, it should be noted that better cognitive functioning after training was based on a combination of physical and cognitive exercise compared with either alone [20].

People are often confronted with situations in daily life that require the concurrent performance of a motor and cognitive task or the concurrent processing of motor and cognitive information such as taking an incoming call when walking or dribbling a ball in basketball. In multi-tasking situations, individuals may have to switch between different task demands or perform two tasks simultaneously. It has also been shown that the performance of competing tasks promotes the activation of widespread areas of the brain, most often the frontal and parietal cortex, as key elements of the distribution of attention during information processing [21,22]. These areas of the brain are known to be the watersheds of the blood supply, at the borders between the vascular pools [23,24]. Chronic ischemia and/or episodes of acute cerebral ischemia during cardiac surgery have been established to have a greater impact on them than any other brain regions. The vulnerability of these regions, not only in the elderly but also in cardiovascular patients, requires professionals to find new approaches to protect and restore brain functions that are associated with them.

The use of a multi-tasking approach, which involves the simultaneous performance of motor and cognitive tasks, may be promising for cognitive rehabilitation in cardiac surgery patients due to the requirement of significant control of attentional and executive functions [25,26]. Previous studies have been reported that simultaneous cognitive–motor training can result in higher benefits in cognitive and motor performance than both training regimes (cognitive or motor training) alone [20]. Although the benefits of a multi-tasking approach have already been demonstrated in patients with Parkinson’s and Alzheimer’s disease, as well as in preventing the risk of falls in the elderly [27,28,29], studies involving cardiac surgery patients are extremely rare. The possibility of using a multi-tasking approach in patients after cardiac surgery is being actively discussed and requires special studies since there is no unequivocal opinion regarding their rehabilitation effect.

It should be taken into account that cardiac surgery patients commonly have a lower functional reserve and a risk of complications in the early postoperative period. It is, therefore, difficult to choose an approach for cognitive recovery due to the physical state of cardiac surgery patients. A multi-tasking approach should include tasks appropriate to their physical condition during the early postoperative period. However, it is also very important to gradually increase the level of difficulty of a cognitive task throughout the learning process. Preliminary evidence suggests that combined motor–cognitive training can provide effective cognitive recovery in patients with cardiovascular disease, optimize cognitive and physical functions, and improve quality of life [30].

Despite the evidence, the multi-tasking approach had not yet been sufficiently implemented into clinical practice. It is necessary to determine the cognitive and motor tasks that require the most activation of the functional reserves of the patients. The optimal training regime and duration of exercises is not certain. We hypothesized that incorporating multi-tasking-based cognitive trainings into the management of patients in the early postoperative period of coronary artery bypass grafting (CABG) will have a positive impact on their cognitive functioning. In addition, we would like to test the benefits of combinations of various cognitive and motor tasks. Thus, the aim of this study was to compare the neuropsychological changes in patients who have undergone two variants of multi-tasking training and a control group in the early postoperative period of coronary artery bypass grafting (CABG).

## 2. Materials and Methods

### 2.1. Data Collection and Sampling

One hundred and ten patients with stable coronary artery disease (CAD) were selected from the cohort of patients of the Cardiology Department Clinic of the Research Institute for Complex Issues of Cardiovascular Diseases. The study was carried out in accordance with the Helsinki Declaration (revised in 2013). The Ethics Committee of the Research Institute for Complex Issues of Cardiovascular Diseases has approved the study (protocol No. 10 dated 10 December 2020). In March 2020, the collection of patient data was initiated. The inclusion criteria were as follows: stable coronary artery disease (CAD), elective CABG, aged 45–75 years, and provided informed consent. The exclusion criteria were as follows: history of stroke, epilepsy, traumatic brain injury, depression, dementia; Montreal Cognitive Assessment Scale (MoCA) score ≤ 18 (30); Beck’s Depression Inventory (BDI-II) score ≥ 8 (31); and non-cardiovascular decompensated comorbidities [30]. All of the patients met the study criteria and signed an informed consent form. Upon admission to hospital, all patients underwent neurological examination, as well as cognitive and depression screening. Reviewers were blinded regarding the participation of patients in the study.

**Figure 1:**
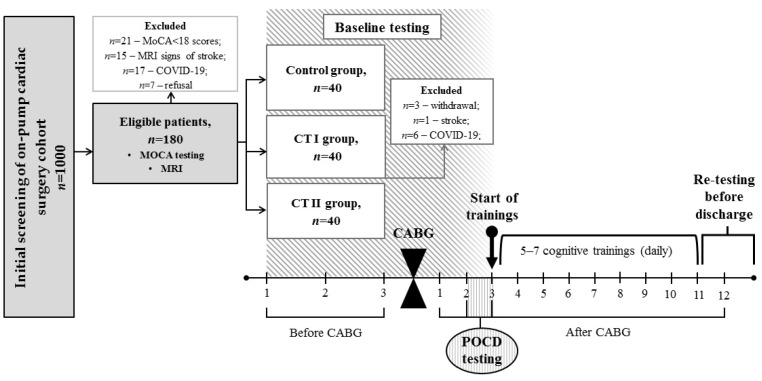
Overview of the study. MOCA, The Montreal Cognitive Assessment; MRI, Magnetic Resonance Imaging; CABG, Coronary Artery Bypass Grafting; POCD, Postoperative Cognitive Dysfunction.

A pseudo-randomization method was used to form three groups, comparable in terms of clinical characteristics. The study sample was divided into the groups: cognitive training (CT) I (*n* = 40), CT II (*n* = 40), and control (*n* = 40). The baseline clinical characteristics are given in Table 1. After initial examination, 10 patients were excluded from the CT I group for various reasons (see Figure 1). The overview of the study design can be seen in Figure 1.

Elective CABG was carried out in all groups using normothermic non-pulsatile cardiopulmonary bypass (CPB). Standard procedures for endotracheal anesthesia and infusion were used. An on-line monitoring of cerebral cortex oxygenation (rSO2) (INVOS-3100, Somanetics, Troy, MI, USA) was carried out. During the surgery time, oxygen saturation indicators were within the normal range. The mean CPB time and surgery time can be found in Table 1. After CABG, all patients were transferred to the intensive care unit for one to two days. The patients were transferred to the cardiology department for postoperative management after intensive care and discharged in 11–12 days.

### 2.2. Neuropsychological Examination

Before study inclusion, the groups of patients (CTI, CT II, and control) were assessed by the screening scale MoCA in the validated Russian-modified version. Extensive neuropsychological testing was used to examine all participants of the study (see Table 2). The baseline testing was carried out 2–3 days before CABG. The first POCD testing was conducted at 2–3 days after surgery. Alternate versions of the neuropsychological tests were used in repeated measurements to minimize practice effects. POCD was determined for each patient individually, using the percentage of relative changes in postoperative indicators compared with a baseline using the following formula: (baseline value–postoperative value)/baseline value) × 100%. Negative values indicated an increase in the cognitive indicator compared to the baseline, positive values indicated a decrease, and the threshold value for cognitive decline was equal to 20% [30]. The examiners were standardized and unaware of the patients’ participation in the study. Upon completion of the cognitive training course or approximately 11–12 days after the CABG, all patients were retested.

### 2.3. Multi-Tasking Training

All cognitive training courses were started 3–4 days after CABG, once daily for a period of 5–7 days. Before the start of the training, the presence of POCD was confirmed according to the above criteria in all patients included in the study. POCD was in 100% of patients in both groups.

### 2.4. CT I (A Postural Task with Mental Arithmetic and Divergent Tasks)

The original multi-tasking training protocol was developed using a postural balance task as a motor subtask, and cognitive subtasks included mental arithmetic, verbal fluency, and divergent tasks. For the postural balance task, trained patients stood on a balance platform to maintain the position of the center of pressure (CoP) at the same point using visual feedback. On the screen of the monitor, the CoP of the subject was presented as a marker, which had to be aligned with the target located in the center of the monitor. Simultaneously to the postural balance, one of the three cognitive tasks was conducted in sequence. The mental arithmetic task involved sequentially subtracting 7 from 100. In the verbal fluency task, the main goal was to produce as many words as possible that start in a given time (60 s). For the divergent task, the participants were asked to generate unusual uses for common objects (e.g., bricks, knives, and newspapers). Each of the cognitive tasks was performed sequentially with resting periods and exit from the balance platform.

### 2.5. CT II (A Simple Visual–Motor Reaction with Mental Arithmetic and Divergent Tasks)

For this training protocol, a simple visual–motor reaction was used as a motor subtask, while the cognitive subtasks were the same as in the CT I protocol. A motor subtask involved pushing the space button as soon as possible upon the appearance of different colors of rectangles on the laptop screen (the number of signals in the test was 30).

The daily training session lasted 20 min, was performed in the morning, and included a preparatory (2 min) and training (10–15 min) period for both CT I and CT II groups. The patient can request to reduce the duration of the training phase. The preparatory period was a discussion with a trainee specialist.

### 2.6. Statistical Analysis

All statistical analyses were conducted using the Statistica 10.0 software (StatSoft, Tulsa, OK, USA, SN: BXXR210F562022FA-A). The distribution of variables was assessed by the Shapiro–Wilk test. Most of the clinical and cognitive variables were not normally distributed. Thus, the parameters are presented as the median with IQR [25th; 75th percentile] and the number of observations (n, %). Continuous variables were evaluated using Kruskal–Wallis one-way analysis of variance and Wilcoxon tests. Continuity-corrected χ^2^ tests were used to determine categorical variables and the percentage relative change in postoperative indicators. The statistical significance of the differences was determined at *p* 0.05.

## 3. Results

### 3.1. Cognitive Performance in CT I

Most patients with CT I training reported an acceptable level of subjective difficulty in performing multi-tasking. Thirty participants completed a course of training. The mean number of training sessions was 5.4. No participants requested a shorter session. The mean time of the training session was 16.0 min at the end of the training course. Cognitive performance changes are presented in Table 3.

As seen in Table 3, there was a statistically significant accelerating speed of psychomotor reactions and an increase in figurative memory after multi-tasking training. In addition, a tendency for an increase in the attention ratio in Bourdon’s test and the attention span test was shown. An individual analysis of cognitive indicators carried out with the cutoff limit of 20% detected multidirectional changes after training. Of the 12 test battery indicators, 2 or more cognitive indicators increased by 20% in 28 patients (93.3%). A combination of improved attention and executive functions was found in 5 patients (16.7%), whereas a combination of improved short-term memory and attention was found in 17 patients (56.7%). An increase in parameters in all domains (psychomotor and executive functions, attention, and short-term memory) was observed in 14 cases (46.7%). Nevertheless, 60% of patients (*n* = 18) met the POCD criteria (a 20% decline in retesting parameters compared to baseline in three cognitive indicators of the test battery).

### 3.2. Cognitive Performance in CT II

The mean number of training sessions was 5.2. No participants requested a shorter session. The mean time of the training session was 15.4 min at the end of the training course. The cognitive performance data of the CT II group are presented in Table 4. Psychomotor speed after multi-tasking training compared to preoperative values was faster, and a tendency for an increase in errors in the executive function test was revealed. There was also an increase in figurative memory compared with preoperative values. An individual analysis of cognitive indicators showed that two or more cognitive indicators increased by 20% in 29 patients (72.5%). A combination of improved attention and executive functions was found in seven patients (17.5%). A combination of improved short-term memory and attention was found only in six patients (15%). An increase in parameters in all domains (psychomotor and executive functions, attention, and short-term memory) was observed in eight cases (20%). Twenty-six patients (65%) met the POCD criteria.

### 3.3. Control Group

At the first POCD testing (2–3 days after surgery), the presence of POCD was confirmed according to the above criteria in all control patients (*n* = 40) included in the study. Before discharge (11–12 days after the CABG), thirty-two patients (80%) met the POCD criteria. An individual analysis of cognitive results revealed that in 25 patients (62.5%), two or more cognitive indicators increased by 20%. Executive functions decreased in 21 patients (52.5%). A combination of short-term memory and attention enhancement was more frequent and was observed in 20 patients (50%). An increase in parameters in all domains (psychomotor and executive functions, attention, and short-term memory) was observed in two cases (5%). The group data of control subjects are presented also in Table 5.

### 3.4. Between-Group Differences

Before surgery, between-group differences in cognitive performance were not observed.

The re-testing revealed that there was only a tendency for differences in POCD incidence between CT I (60%) and the control group (80%) at 11–12 days after the CABG: OR = 2.7; 95% CI: 0.92–7.73; z statistic = 1.805; *p* = 0.07. Additionally, CT II (65%) differed from the control patients (80%) not significantly: OR = 2.2; 95% CI: 0.78–5.92; z statistic = 1.487; *p* = 0.14.

**Figure 2:**
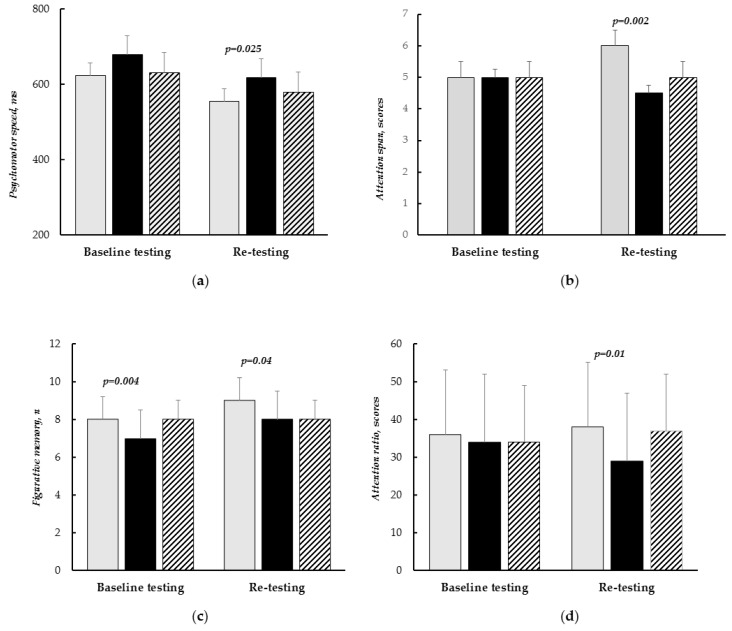
Between-group differences in cognitive performance after multi-tasking training (re-testing). (**a**)—psychomotor speed; (**b**)—attention span; (**c**)—figurative memory; (**d**)—attention ratio. Light bars—CT I patients; dark bars—CT II, shaded bars—control group. Data are presented as the median with IQR [25th; 75th percentile].

Between-group differences in cognitive performance after multi-tasking training and re-testing are illustrated in Figure 2. This analysis (see Figure 2a) revealed the differences in psychomotor speed with lower values in the CT II group compared to CT I. The control group did not differ with regard to psychomotor speed from CT I and CT II groups.

In addition, differences between CT I and CT II groups were in figurative memory (Figure 2c) and attention indicators (Figure 2b,d). These differences were due to better cognitive performance in CT I patients compared to CT II after multi-tasking training. The results of control patients were the same with CT II and lower compared with CT I, but there were no significant differences.

As a result of an individual analysis of cognitive performance, it was established that CT I patients demonstrated an improvement of two or more cognitive indicators in a larger number of cases (93.3% vs. 72.5%), OR = 5.31, 95% CI = 1.1–26.1, Z = 2.05, *p* = 0.04, as compared to the CT II group. There were significant differences in the improvement of two or more cognitive indicators (93.3% vs. 62.5%), OR = 8.40, 95% CI = 1.75–40.41, Z = 2.66, *p* = 0.008, between CT I and the control group.

In addition, the combination of improved short-term memory and attention was found more frequently in the CT I group compared to CT II (56.7% vs. 15%), OR = 7.41, 95% CI = 2.4–22.9, Z = 3.48, *p* = 0.0005. There was no difference between the CT I and control patients in the combination of improved short-term memory and attention (56.7% vs. 50%), OR = 1.31, 95% CI = 0.5–3.4, Z = 0.55, *p* = 0.58. CT II patients demonstrated the combination of improved short-term memory and attention in a fewer number of cases (15% vs. 50%) in comparison to control patients, OR = 0.18, 95% CI = 0.6–0.51, Z = 3.19, *p* = 0.001. The cognitive improvement of all domains (psychomotor and executive functions, attention, and short-term memory) was also revealed in CT I patients more frequently compared with CT II and control groups (46.7% vs. 20%, OR = 3.5, 95% CI = 3.38–81.74, Z = 2.33, *p* = 0.02 and 46.7% vs. 5%, OR = 16.63, 95% CI = 1.22–10.1, Z = 3.46, *p* = 0.0005), respectively.

## 4. Discussion

The study results showed positive effects of the multi-tasking training on cognitive functions in cardiac surgery patients. In 93.3% of cases in CT I patients, there was an improvement in two or more cognitive indicators, which is significantly higher than in the CT II group (72.5%). In addition, the CT I group demonstrated the improvement of two or more cognitive indicators in a larger number of cases in comparison with control patients (93.3% vs. 62.5%). A combination of improved short-term memory and attention was also found more frequently in the CT I group compared to the CT II patients (56.7% vs. 15%). However, the cognitive improvement of all domains was in a larger number of cases in the CT I and CT II patients in comparison to the control group (46.7% and 20% vs. 5%). This was shown by an individual analysis of cognitive indicators.

The advantages of the CT I multi-tasking approach were also demonstrated in comparison to CT II in the results of between-group comparisons. CT I patients had better figurative memory, psychomotor speed, and attention indicators than CT II after multi-tasking training. Thus, the combination of a postural task with mental arithmetic and divergent tasks has a more beneficial effect on cognitive performance in cardiac surgery patients.

As shown previously, a successful option for multi-tasking may be a combination of motor training and attention or executive function tasks [16,31,32]. Some authors have pointed to the positive effect of their inclusion in the rehabilitation course [28,29,30]. It was also reported that these elements have a positive effect on cognitive abilities and recommend a cautious increase in task complexity, depending on the individual abilities of the subject [31]. However, this applies to cognitively and clinically intact individuals. The early postoperative period with pain syndrome, asthenia, and other surgical complications can restrict the physical status of cardiac surgery patients. Therefore, the motor component selection for the dual task is limited. We opted for postural training and a simple visual–motor task to ensure maximum tolerance in the difficult cohort of patients. Dual tasks including postural training were previously used to restore cognitive function in traumatic brain damage [33]. In fact, in our study, the training with a postural motor task (CT I) demonstrated better results than CT II with a simple visual–motor reaction task.

It should be noted, however, that the multi-tasking training conducted in the early postoperative period of CABG had a limited impact on the cognitive performance in the patients of both groups in terms of POCD incidence. The frequency of POCD was quite high (60% in CT I and 65% in CT II). According to the literature, POCD frequency can reach 70–80% [12,13,14]. Our previous study found that patients who underwent standard recovery therapy had a POCD frequency of 79.5% in the early postoperative period of cardiac surgery [14].

It was shown that the performance of each of the components of the dual task can be impaired by interference processes [28,33,34]. In our study, the insignificant clinical effect of the multi-tasking paradigms chosen can be explained by the interference interactions between cognitive and motor components. It has been demonstrated that for older people, performing competitive tasks causes cognitive component deterioration in a combination of complex cognitive tasks and any motor task, and motor tasks are performed less by them combined with complex cognitive tasks [35]. Since the divergent task used in this study as a cognitive component can be classified as a complex cognitive task, the limited cognitive resources of patients in the early postoperative period of CABG did not allow them to cope effectively with the proposed version of the training.

Multi-tasking difficulties have been established in the aging population [21,36,37]. A number of studies have also shown interference effects during the concurrent performance of motor tasks involving postural control and cognitive tasks, with pronounced effects in the elderly [32,38,39]. The study of Brahms et al. [40] investigated the effects of cognitive-motor multi-tasking interventions on postural stability and cognitive performance in healthy older adults. These findings showed that the simultaneous performance of cognitive and postural tasks was moderated by modality compatibility mapping, workload memory, and increased postural demands. However, postural and cognitive performance did not change as a result of training. Bohle and his colleagues showed an age-related decline in cognitive performance at high cognitive–postural task demands [41].

Nevertheless, the CT I version of multi-tasking cognitive training can be effective in improving the cognitive state of cardiac surgery patients, as it provides a greater transfer effect (improvement in short-term memory and attention in the post-training period compared to the baseline). Previously, it was found that the effect of the transfer varies depending on the complexity and modality of the task performed [42]. It is suggested that the effectiveness of the multi-tasking approach in the recovery of cognitive functions is ensured by more effective coordination of cognitive processes.

It should also be noted that, in this study, multi-tasking training was used as a short course (5–7 training sessions). The obtained data indicate the formation of beneficial effects on cognitive functions in a short time period after CABG, which is especially important in patients to establish medical adherence and optimization of rehabilitation procedures in general. In most previous studies, short-term effects of multi-tasking training were not studied, especially in the cardiac cohort of patients [40,41,43]. It can be assumed that if the duration of the course is extended, it would have a greater positive impact on cognitive performance in cardiac surgery patients. Therefore, future studies should separately consider the impact of the length of the course of multi-tasking training on the involvement of different cognitive domains. The sustainability of the positive effects of short-term cognitive training on the preservation of the patient’s overall intellectual functions also requires careful study. It is necessary to further improve approaches to multi-tasking postoperative training with an intensification load and individual support for cardiac surgery patients in the long-term postoperative period.

New technologies may offer new opportunities for medical research and practice, including virtual reality (VR) [15,44,45]. The manipulation of experimental parameters in VR software has great potential for new forms of intervention and treatment of cognitive and motor disorders in patients with different pathology, including ischemic brain damage. Further studies in the field of adapting successful multi-tasking trainings to the VR interface to the effective training of memory, executive functioning, and attention are needed.

## 5. Limitations

When interpreting the findings, it is important to take into account the limitations of our study. The study’s observational nature was a limitation, and the effectiveness of CT for patients was assessed through an individual analysis of cognitive performance. Additionally, only a short early postoperative period of CABG was used for training. The other limitation was the small sample of patients, as we only recruited consecutive ones. Thus, we performed this pilot study to plan a larger and more comprehensive prospective study.

## 6. Conclusions

The CT I multi-tasking training was more effective in improving the cognitive performance in the early postoperative period of CABG in comparison to CT II training and standard post-surgery management of patients. The combination of a postural task with mental arithmetic and divergent tasks provided a greater transfer effect (better results in short-term memory and attention). The findings of this study raise important questions regarding the effectiveness of multi-tasking interventions and will be helpful for designing and implementing future studies involving multi-tasking training. Consequently, future studies should investigate interventions with different lengths of the multi-tasking training course in larger samples.

## Figures and Tables

**Table 1:** The clinical and anamnestic characteristics of all patients before cardiac surgery (*n* = 110).

Variable	Cognitive Training I (*n* = 30)	Cognitive Training II (*n* = 40)	Control (*n* = 40)	*p*-Value
Age, years, Me [Q25; Q75]	65 [60; 68]	65.5 [61; 70.5]	65 [61; 69]	0.37 *
MoCA, scores, Me [Q25; Q75]	25 [22; 26]	25 [24; 27]	26 [23; 27]	0.65 *
BDI-II, scores, Me [Q25; Q75]	5 [2; 6]	3 [2; 4]	4 [1; 5]	0.08 *
Educational attainment, years, Me [Q25; Q75]	12 [11; 16]	11 [10; 15]	12 [10; 15]	0.11 *
Functional class of angina, *n* (%) I–II III	26 (86.7) 4 (13.3)	29 (72.5) 11 (27.5)	31 (77.5) 9 (22.5)	0.41 ^#^
Functional class NYHA, *n* (%) I–II III	27 (90) 3 (10)	39 (97.5) 1 (2.5)	37 (92.5) 3 (7.5)	0.46 ^#^
History of myocardial infarction, *n* (%)	16 (53.3)	18 (45)	27 (67.5)	0.13 ^#^
Fraction of left ventricle ejection, %, Me [Q25; Q75]	62 [52; 68]	64 [52.5; 66]	64 [58; 67]	0.81 ^#^
Type 2 of diabetes mellitus, *n* (%)	9 (30)	10 (25)	16 (40)	0.65 ^#^
CA stenosis < 50%, *n* (%)	17 (56.7)	22 (55)	12 (30)	0.14 ^#^
Cardiopulmonary bypass time, min, Me [Q25; Q75]	85 [68; 102]	81 [68; 99]	72 [56; 103]	0.45 *
Surgery time, min, Me [Q25; Q75]	225 [175; 241]	220 [180; 245]	200 [180; 228]	0.49 *
Medication, *n* (%)				
ACEi	16 (53.3)	28 (70)	27 (67.5)	0.28 ^#^
Statins	30 (100)	40 (100)	40 (100)	-
Beta-blockers	30 (100)	38 (95)	38 (95)	0.49 ^#^
Antiplatelet drugs	30 (100)	40 (100)	40 (100)	-
CCB	7 (23.3)	10 (25)	11 (27.5)	0.53 ^#^
ARB	7 (23.3)	9 (22.5)	8 (20)	0.91 ^#^
Diuretics	24 (80)	32 (80)	30 (75)	0.80 ^#^

ACEi, angiotensin-converting enzyme inhibitor; ARBs, angiotensin II receptor blockers; CA, carotid artery; CCB, calcium channel blocker; NYHA, heart failure according to the New York Heart Association. *—between-group differences by Kruskal–Wallis one-way analysis of variance; ^#^—between-group differences by χ^2^.

**Table 2:** Cognitive test battery for assessing cognitive function in cardiac surgery patients.

Cognitive Tests and Indicators	Description of the Procedure
Cognitive screening	
Montreal Cognitive Assessment Scale (MoCA), scores	30-point questionnaire for cognitive impairment and dementia screening (Russian-modified version).
Psychomotor and executive functions	
Complex visual–motor reaction Reaction time, ms Errors, *n*	Reaction latencies of the right and left hands to stimuli (different colors of rectangles) when the subject can choose one of the three presented signals (the number of signals in the test is 30).
Level of functional mobility of nervous processes: responses to feedback Reaction time, ms Errors, *n* Missed signals, *n*	Feedback mode is used for the performance of the previous test. The exposure time of the test signals (rectangles) is changed: the exposure of the next signal is shortened by 20 ms with each correct answer and extended by 20 ms with an incorrect answer. The test contains 120 signals. A missed signal is indicated by the absence of response to the test signal.
Attention	
The Bourdon’s test Processed letters per min, *n* Processed letters per 4 min, *n* Attention ratio, scores	The subject is provided with the alphabetic version of Bourdon’s test to highlight certain letters for a time of 4 min.
Attention span, scores	The subject is presented with a square grid of 16 equal cells. Crosses appear in different parts of the grid for a short time, and the subject must memorize their location and mark the corresponding cells with the left mouse button immediately after the stimulus disappears.
Short-term memory	
10 words memorizing test, *n*	To remember as many of 10 words presented one after another as possible.
10 numbers memorizing test, *n*	To remember as many of 10 numbers presented one after another as possible.
Figurative memory, *n*	The subject is presented with 10 figures, which must be remembered (30 s memory time). Next appears a set of 30 figures, among which it is necessary to find and mark with the left mouse button all previously remembered figures.

**Table 3:** Cognitive performance changes in CT I patients.

Cognitive Indicator	Baseline Testing (*n* = 30)	Retesting (after Training) (*n* = 30)	*p*
Complex visual–motor reaction			
Reaction time, ms Errors, *n*	622.5 [584; 703] 1 [1; 2]	555 [512; 601] 1 [1; 2]	0.000005 0.84
Level of functional mobility of nervous processes: responses to feedback			
Reaction time, ms Errors, *n* Missed signals, *n*	478.5 [447; 511] 28 [23; 31] 15 [10; 21]	475 [456; 516] 26 [24; 30] 13 [8; 20]	0.5 0.51 0.84
Attention			
Bourdon’s test			
Processed letters per min, *n* Processed letters per 4 min, *n* Attention ratio, scores	71 [48; 89] 102 [78; 117.5] 36 [28; 48]	64 [51; 86] 102 [75; 117] 38 [30; 53]	0.4 0.1 0.08
Attention span test, scores	5 [4; 8]	6 [5; 7]	0.27
Short-term memory			
Figurative memory test, *n*	8 [6; 9]	9 [8; 10]	0.0004
10 words memorizing test, *n*	4 [4; 5]	4 [4; 5]	0.82
10 numbers memorizing test, *n*	4 [3; 5]	4 [3; 6]	0.1

Data are presented as the median with IQR [25th; 75th percentile].

**Table 4:** Cognitive performance changes in CT II patients.

Cognitive Indicator	Baseline Testing (*n* = 40)	Retesting (after Training) (*n* = 40)	*p*
Complex visual–motor reaction			
Reaction time, ms Errors, *n*	677.5 [621; 756.5] 1 [0; 1,5]	618 [547.5; 684] 1 [0.5; 3]	0.0001 0.11
Level of functional mobility of nervous processes responses to feedback			
Reaction time, ms Errors, *n* Missed signals, *n*	493.5 [473; 528] 26 [21; 29] 17.5 [10; 20.5]	486 [457; 536] 25.5 [23; 29.5] 14 [11; 19]	0.44 0.46 0.77
Attention			
Bourdon’s test			
Processed letters per min, *n* Processed letters per 4 min, *n* Attention ratio, scores	65 [48; 79] 78 [57; 127] 34 [29; 47]	57.5 [47; 80] 80 [58; 111] 29 [26; 39]	0.25 0.65 0.04
Attention span test, scores	5 [4; 6]	4.5 [4; 5.5]	0.32
Short-term memory			
Figurative memory test, *n*	7 [6; 8]	8 [7; 9]	0.007
10 words memorizing test, *n*	4 [3; 5,5]	4 [3; 5]	0.86
10 numbers memorizing test, *n*	4 [3; 5,5]	4 [4; 5]	0.93

Data are presented as the median with IQR [25th; 75th percentile].

**Table 5:** Cognitive performance changes in the control group of patients.

Cognitive Indicator	Baseline Testing (*n* = 40)	Retesting (11–12 Days after the CABG) (*n* = 40)	*p*
Complex visual–motor reaction			
Reaction time, ms Errors, *n*	631 [556; 684] 1 [0; 2]	579 [536; 635] 1 [1; 3]	0.0007 0.12
Level of functional mobility of nervous processes: responses to feedback			
Reaction time, ms Errors, *n* Missed signals, *n*	488 [455.5; 521.5] 25 [22.5; 29] 16 [10; 23]	488 [453.5; 542.5] 28.5 [24; 29.5] 14 [9; 18]	0.23 0.05 0.1
Attention			
Bourdon’s test			
Processed letters per min, *n* Processed letters per 4 min, *n* Attention ratio, scores	66 [42; 99] 82 [69; 112.5] 34 [27; 47]	68 [49.5; 89.5] 90 [67; 109.5] 37 [27; 50]	0.43 0.69 0.15
Attention span test, scores	5 [4; 7]	5 [4; 6]	0.96
Short-term memory			
Figurative memory test, *n*	8 [6; 9]	8 [6.5; 9]	0.14
10 words memorizing test, *n*	4.5 [3; 5]	4 [4; 5]	0.35
10 numbers memorizing test, *n*	4 [3; 6]	4 [3; 5]	0.86

Data are presented as the median with IQR [25th; 75th percentile].

## Data Availability

Not applicable.
